# Secondary Metabolites with Antioxidant and Antimicrobial Activities from *Camellia fascicularis*

**DOI:** 10.3390/cimb46070404

**Published:** 2024-07-02

**Authors:** Jiandong Tang, Ruonan Li, Boxiao Wu, Junrong Tang, Huan Kan, Ping Zhao, Yingjun Zhang, Weihua Wang, Yun Liu

**Affiliations:** 1 Key Laboratory of Forest Resources Conservation and Utilization in the Southwest Mountains of China Ministry of Education, Southwest Forestry University, Kunming 650224, China; hdyknctjd23@swfu.edu.cn (J.T.); 15912417317@163.com (R.L.); wbx1437@swfu.edu.cn (B.W.); tjrzy2016@swfu.edu.cn (J.T.); kanhuan@swfu.edu.cn (H.K.); hypzhao2023@163.com (P.Z.); 2 State Key Laboratory of Phytochemistry and Plant Resources in West China, Kunming Institute of Botany, Chinese Academy of Sciences, Kunming 650224, China; zhangyj@mail.kib.ac.cn

**Keywords:** *Camellia fascicularis*, chemical composition, antioxidant, antimicrobial

## Abstract

*Camellia fascicularis* has important ornamental, medicinal, and food value. It also has tremendous potential for exploiting bioactivities. However, the bioactivities of secondary metabolites in *C. fascicularis* have not been reported. The structures of compounds were determined by spectral analysis and nuclear magnetic resonance (NMR) combined with the available literature on secondary metabolites of *C. fascicularis* leaves. In this study, 15 compounds were identified, including 5 flavonoids (**1**–**5**), a galactosylglycerol derivative (**6**), a terpenoid (**7**), 4 lignans (**8**–**11**), and 4 phenolic acids (**12**–**15**). Compounds **6**–**7** and **9**–**12** were isolated from the genus *Camellia* for the first time. The remaining compounds were also isolated from *C. fascicularis* for the first time. Evaluation of antioxidant and antimicrobial activities revealed that compounds **5** and **8**–**11** exhibited stronger antioxidant activity than the positive drug ascorbic acid, while compounds **7**, **13**, and **15** showed similar activity to ascorbic acid. The minimum inhibitory concentration (MIC) of antibacterial activity for compounds **5**, **7**, **9**, **11**, and **13** against *Pseudomonas aeruginosa* was comparable to that of the positive control drug tetracycline at a concentration of 62.50 µg/mL; other secondary metabolites inhibited *Escherichia coli* and *Staphylococcus aureus* at concentrations ranging from 125–250 µg/mL.

## 1. Introduction

*Camellia fascicularis*, a genus of *Camellia* in the family Theaceae, is an endemic plant in Yunnan province, China. *C. fascicularis*, which is a rare species resource with unique golden petals, also known as “giant panda in the plant kingdom”, “queen of the tea family”, and “living fossil of plants” was first discovered in Hekou County and only distributed in Gejiu, Maguan, and Hekou counties [1]. *C. fascicularis* leaves possess high amino acid and mineral content and are considered an edible plant resource with high nutritional and health value [2,3]. There is limited literature on the metabolites of *Camellia*, with polyphenols, flavonoids, saponins, and steroids being the primary active components involved [4,5,6,7,8]. It has been proved by experiments that the flowers and leaves of *C. fascicularis*, in addition to being used as a tea, have many pharmacological effects such as antioxidant [9], anti-tumor [10,11], and anti-inflammatory [12,13] attributes. Due to its limited distribution in Yunnan, the investigation into secondary metabolites of *C. fascicularis* commenced relatively late; nevertheless, it harbors immense potential for exploring its biological activities.

The isolation and identification of monomeric active compounds from complex plant components is a pivotal objective in the field of natural product chemistry research [14]. However, the bioactivities of secondary metabolites in *C. fascicularis* have not been reported. Considering the significance of previous research findings in exploring the bioactivities of *C. fascicularis*, this study aimed to investigate the secondary metabolites and bioactivities (antioxidant and antimicrobial) present in the acetate fraction of the methanol extract derived from *C. fascicularis* leaves. This investigation facilitated a comprehensive understanding of the composition of monomeric compounds within *C. fascicularis* leaves, while also anticipating the isolation of novel compounds with remarkable bioactive properties.

## 2. Materials and Methods

### 2.1. Instrumentation

The following instruments were used in the study: Bruker AV 500 MHz Nuclear Magnetic Resonance Instrument, (Bruker, Saarbrucken, Germany), XEVO G2-XS Q-Tof High Resolution Mass Spectrometer (Waters, Milford, MA, USA), NP7000 Semi-preparative Liquid Phase (Jiangsu Hanbang Technology Co., Ltd., Huaian, China), AX224ZH\E Electronic Balance (Ohaus Instruments, Changzhou Co., Ltd., Changzhou, China), N-1300 rotary evaporator, CA-111 cold trap (Shanghai Ailang Instrument Co., Ltd., Shanghai, China), SHZ-DⅢ circulating water vacuum pump (Gongyi Yuhua Instrument Co., Ltd., Gongyi, China), SpectraMax 190 enzyme labeler (Molecular Devices Co., Ltd., Shanghai, China), ZQZY-CF9.9 oscillating incubator (Shanghai Zhichu Instrument Co., Ltd., Shanghai, China), and a ZF-7 triple-use UV analyzer (Shanghai Jiapeng Science and Technology Co., Ltd., Shanghai, China).

### 2.2. Chemicals and Reagents

2,2’-azino-bis(3-ethylbenzothiazoline-6-sulfonic acid) (ABTS) and ascorbic acid were obtained from Beijing Solarbio Science & Technology Co., Ltd. (Beijing, China). Methanol (HPLC grade) was purchased from Shanghai Xingke High Purity Solvent Co., Ltd. (Shanghai, China). Dimethyl sulfoxide (DMSO), and all other chemicals of analytical grade were purchased from Sinopharm Chemical Reagent Co., Ltd. (Shanghai, China). Macroporous resin D101 and Sephadex LH–20 were purchased from Shanghai Yuanye Biotechnology Co., Ltd. (Shanghai, China). Column chromatography silica gel (200–300 mesh and 300–400 mesh) and thin-layer chromatography silica gel plates were purchased from Qingdao Ocean Chemical Co., Ltd. (Qingdao, China), and middle chromatogram isolated (MCI) was purchased from Beijing Lvbaicao Technology Development Co., Ltd. (Beijing, China). The reagents (industrial grade) used in the column chromatography process were purchased from Yunnan Liyan Technology Co Ltd. (Kunming, China).

### 2.3. Plant Material

The voucher specimen (52,860) of *C. fascicularis* was identified by taxonomist Min Tianlu and preserved in the Herbarium of Kunming Institute of Botany, Chinese Academy of Sciences. The leaves of *C. fascicularis* used in this experiment were obtained from Dawei Mountain Nature Reserve, Hekou County, Yunnan Province, China, in December 2019, and were identified as *C. fascicularis* by taxonomist Prof. Xiang Jianying of Southwest Forestry University.

### 2.4. Extraction and Isolation

The 10.7 kg of dried *C. fascicularis* samples were crushed to about 40 mesh and extracted with 95% methanol (100 L) with stirring assistance at 50 °C for 3, 2, and 1 h, respectively. The extracts were combined and the solvents were evaporated under low pressure at 50 °C. The methanol extract of *C. fascicularis* (868.3 g) was ultimately obtained. Subsequently, 5 L of distilled water was added for thorough ultrasonic mixing, followed by three extractions using equal volumes (5 L) of industrial ethyl acetate. The ethyl acetate phase extract (177.8 g) was obtained via low-pressure rotary evaporation at 40 °C, and subsequently mixed with macroporous resin (267.0 g) before being loaded onto a column and eluted using a MeOH:H_2_O gradient (0:1→1:0). The resulting fractions were combined based on thin-layer chromatography detection, yielding 4 distinct fractions (Fr. I–IV). The combined fraction of (Fr. II–III) from the extract was subjected to silica gel column chromatography, employing a gradient elution of CHCl_3_:MeOH (50:1, 30:1, 15:1, 10:1, 5:1, 2:1, 1:1, *v*/*v*), resulting in the isolation of 8 fractions (Table 1).

Fr. G was eluted with a CHCl_3_:MeOH = 2:1, *v*/*v* and yielded 9.0 g. It was separated by MCI reversed-phase column chromatography and gradient elution with MeOH–H_2_O (2:3, 1:1, 3:2, 7:3, 4:1, 9:1, 10:0, *v*/*v*) to obtain 9 flow fractions (Ga–Gi). Fr. Gd was further purified and impurities removed using Sephadex LH–20 (CH_2_Cl_2_:MeOH = 1:1), followed by separation by elution with small orthophase silica gel (CH_2_Cl_2_:MeOH), and finally purified using preparative high-performance liquid chromatography (PHPLC) to give monomeric compound **1** (3.1 mg, V_methanol:water_ = 58:42, *t*_R_ = 8 min), **2** (3.2 mg, V_methanol:water_ = 58:42, *t*_R_ = 10 min), and **3** (5.3 mg, V_methanol:water_ = 59:41, *t*_R_ = 7 min). Fr. Ge was further purified and decontaminated using Sephadex LH–20 (CH_2_Cl_2_:MeOH = 1:1) to give compound **4** (13.0 mg) and **5** (5.6 mg). Fr. Gi was further purified and impurities removed using Sephadex LH–20 (CH_2_Cl_2_:MeOH = 1:1), followed by separation by elution with small orthophase silica gel (CH_2_Cl_2_:MeOH), and finally purified using PHPLC to give monomeric compound **6** (7.0 mg, V_methanol:water_ = 83:17, *t*_R_ = 22 min).

Fr. A was eluted with a CHCl_3_:MeOH = 50:1, *v*/*v* and yielded 6.0 g. It was separated by MCI column chromatography and gradient elution with MeOH–H_2_O (2:3, 1:1, 3:2, 7:3, 4:1, 9:1, 10:0, *v*/*v*) to obtain 9 flow fractions (Aa–Ai). Fr. Ab was further purified and impurities removed using Sephadex LH–20 (MeOH:H_2_O = 95:5) and finally purified using PHPLC to give monomeric compound **7** (5.8 mg, V_methanol:water_ = 27:73, *t*_R_ = 25 min). Fr. Ae was further purified and impurities removed using Sephadex LH–20 (MeOH:H_2_O = 95:5) and finally purified using PHPLC to give monomeric compound **8** (29.1 mg, V_methanol:water_ = 54:46, *t*_R_ = 18 min), **9** (4.8 mg, V_methanol:water_ = 54:46, *t*_R_ = 20 min), **10** (4.3 mg, V_methanol:water_ = 54:46, *t*_R_ = 22 min), and **11** (5.7 mg, V_methanol:water_ = 54:46, *t*_R_ = 25 min). Fr. Aa was further purified and impurities removed using Sephadex LH–20 (MeOH:H_2_O = 95:5) and finally purified using PHPLC to give monomeric compound **12** (29.1 mg, V_methanol:water_ = 30:70, *t*_R_ = 12 min), **13** (4.8 mg, V_methanol:water_ = 30:70, *t*_R_ = 14 min), **14** (4.3 mg, V_methanol:water_ = 30:70, *t*_R_ = 16 min), and **15** (7.2 mg, V_methanol:water_ = 30:70, *t*_R_ = 16 min).

**Figure 1:**
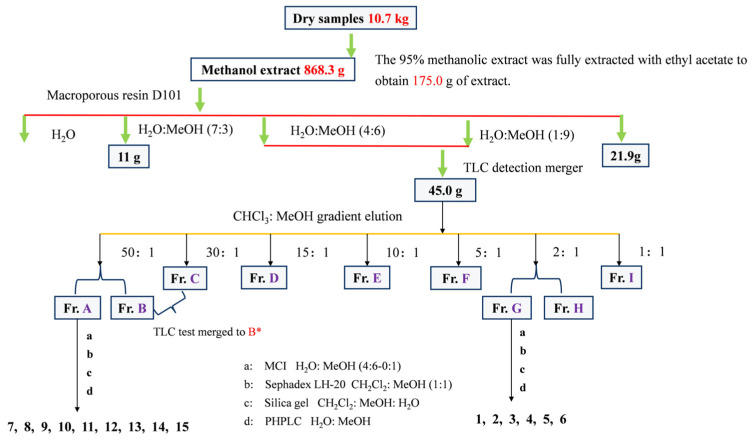
Flow chart for the isolation and purification of compounds **1**–**15**.

After obtaining the monomeric compounds through purification, the organic solvent was evaporated and quantified. A suitable deuterium substitute reagent was selected for dissolution and sent for testing, with TMS used as an internal standard. The isolation and purification process of compounds 1–15 is illustrated in Figure 1.

### 2.5. Chemical Structure Analysis

**Figure 2:**
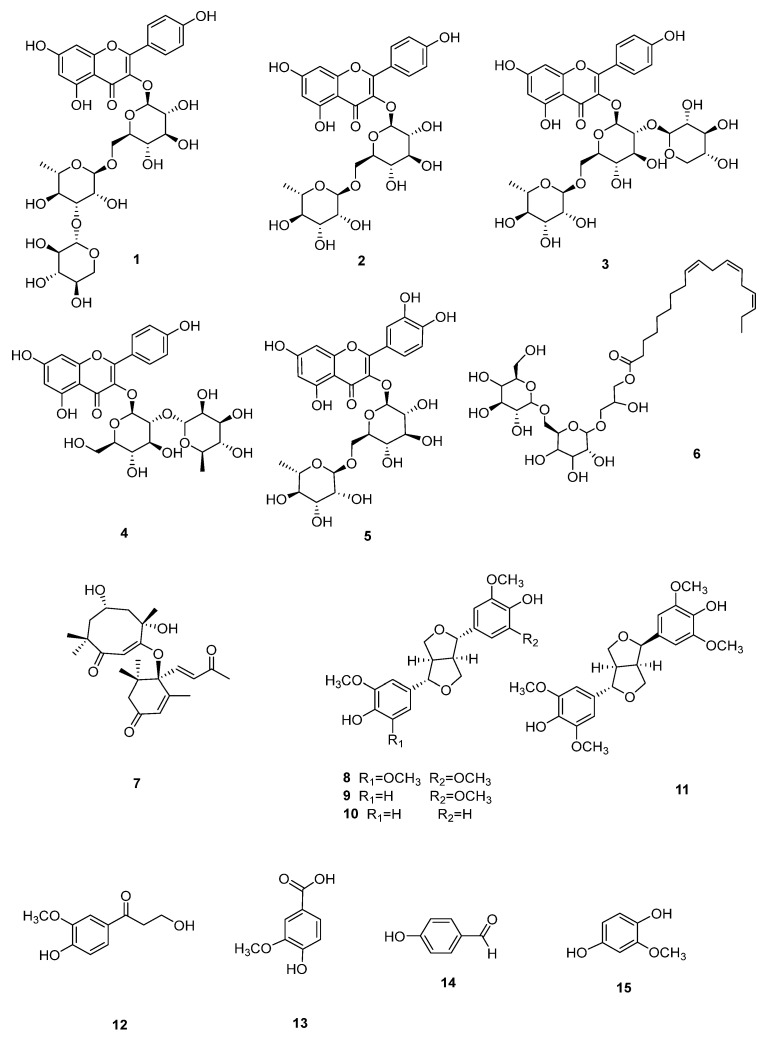
Structures of compounds **1**–**15**.

Secondary metabolites investigation on the leaves of *C. fascicularis*, afforded 15 compounds, including 5 flavonoids (**1**–**5**), a galactosylglycerol derivative (**6**), a terpenoid (**7**), 4 lignans (**8**–**11**), and 4 phenolic acids (**12**–**15**). The structures of these compounds were established via spectroscopic analysis and comparison of their NMR data with the literature. Compounds **6**–**7** and **9**–**12** were isolated from the genus *Camellia* for the first time. The remaining compounds were also isolated from this plant for the first time. The structures of the known compounds (Figure 2).

Tsubakioside A (**1**): Pale yellow powder, HRESIMS *m/z* 749.1808 [M + Na]^+^ (calcd. for C_32_H_38_O_19_); ^1^H NMR (500 MHz, Methanol-*d*_4_) *δ*_H_ 8.06 (2H, d, *J* = 8.6 Hz, H-2’ and 6’), 6.89 (2H, d, *J* = 8.6 Hz, H-3’ and 5’), 6.41 (1H, d, *J* = 2.0 Hz, H-8), 6.21 (1H, d, *J* = 2.0 Hz, H-6), 5.12 (1H, d, *J* = 7.4 Hz, Glc H-1), 4.51 (1H, d, *J* = 1.4 Hz, Rha H-1), 4.34 (1H, d, *J* = 7.5 Hz, Xyl H-1), 3.84–3.18 (15H, m), 1.10 (3H, d, *J* = 6.1 Hz, Rha 5-CH_3_). ^13^C NMR (126 MHz, Methanol-*d*_4_) *δ*_C_ 179.7 (C-4), 166.9 (C-7),163.4 (C-5), 161.9 (C-4’), 159.8 (C-9), 159.0 (C-2), 135.8 (C-3), 132.8 (C-2’ and 6’), 123.2 (C-1’), 116.5 (C-3’ and 5’), 106.8 (Xyl C-1), 105.9 (C-10), 104.8 (Glc C-1), 102.7 (Rha C-1), 100.5 (C-6), 95.5 (C-8), 82.8 (Rha C-3), 78.5 (Glc C-3), 77.9 (Glc C-5), 77.6 (Xyl C-3), 76.1 (Glc C-2), 75.6 (Xyl C-2), 73.0 (Rha C-4), 72.1 (Rha C-4), 72.0 (Rha C-2), 71.4 (Glc C-4), 71.4 (Xyl C-4), 69.8 (Rha C-6), 69.3 (Glc C-6), 67.2 (Xyl C-5), 18.3 (Rha C-6). The structures of these compounds were established via spectroscopic analysis and comparison of their NMR data with the literature [15].

Kaempferol 3-*O*-rutinoside (**2**): Yellow amorphous powder, HRESIMS *m/z* 617.1494 [M + Na]^+^ (calcd. for C_27_H_30_O_15_); ^1^H NMR (500 MHz, Methanol-*d*_4_) *δ*_H_ 8.05 (2H, d, *J* = 9.1 Hz, H-2″, 6″), 6.88 (2H, d, *J* = 8.7 Hz, H-3″, 5″), 6.41 (1H, d, *J* = 2.1 Hz, H-8), 6.22 (1H, d, *J* = 2.1 Hz, H-6), 5.13 (1H, d, *J* = 7.5 Hz, Glc H-1), 4.51 (1H, d, *J* = 1.4 Hz, Rha H-1), 3.82–3.32 (10H, m, Glc H-2, 6 and Rha H-2, 5), 1.12 (3H, d, *J* = 6.2 Hz, Rha H-6). ^13^C NMR (126 MHz, Methanol-*d*_4_) *δ*_C_ 179.4 (C-4), 166.2 (C-7), 163.0 (C-5), 161.5 (C-4’), 159.4 (C-9), 158.6 (C-2), 135.5 (C-3), 132.4 (C-2’, 6’), 122.8 (C-1’), 116.2 (C-3’, 5’), 105.7 (C-10), 104.6 (C-1″), 102.4 (C-1‴), 100.0 (C-6), 94.9 (C-8), 78.2 (C-3″), 77.2 (C-2″), 75.8 (C-5″), 73.9 (C-4‴), 72.3 (C-2‴), 72.1 (C-3‴), 71.5 (C-4″), 69.7 (C-5‴), 68.6 (C-6″), 17.9 (C-6‴). The structures of these compounds were established via spectroscopic analysis and comparison of their NMR data with the literature [16].

Camelliaside B (**3**): Pale yellowish powder, HRESIMS *m/z* 749.1808 [M + Na]^+^ (calcd. for C_32_H_38_O_19_); ^1^H NMR (500 MHz, Methanol-*d*_4_) *δ*_H_ 8.07 (2H, d, *J* = 8.8 Hz, H-2’, 6’), 6.89 (2H, d, *J* = 8.6 Hz, H-3’, 5’), 6.41 (1H, d, *J* = 2.0 Hz, H-8), 6.22 (1H, d, *J* = 2.0 Hz, H-6), 5.12(1H, d, *J* = 7.4 Hz, Glc H-1), 4.51 (1H, d, *J* = 1.5 Hz, Rha H-1), 4.34 (1H, d, *J* = 7.6 Hz, Xyl H-1), 4.25–3.09 (15H, m), 1.10 (3H, d, *J* = 6.1 Hz, Rha 5-CH_3_). ^13^C NMR (126 MHz, Methanol-*d*_4_) *δ*_C_ 179.1 (C-4), 164.9 (C-7), 162.7 (C-5), 161.2 (C-4’), 159.1 (C-9), 158.3 (C-2), 135.1 (C-3), 132.1 (C-2’, 6’), 122.4 (C-1’), 115.8 (C-3’, 5’), 106.1 (C-10), 105.3 (Xyl C-1), 102.0 (Rha C-1), 100.2 (Glc C-1), 99.7 (C-6), 94.7 (C-8), 82.1 (Glc C-2), 77.8 (Glc C-3), 77.2 (Xyl C-3), 76.9 (Glc C-5), 75.4 (Xyl C-2), 74.3 (Rha C-4), 72.3 (Rha C-3), 72.2 (Rha C-2) 71.4 (Glc C-4), 70.8 (Xyl C-4),69.1 (Rha C-5), 68.6 (Glc C-6), 66.5 (Xyl C-5), 17.6 (Rha C-6). The structures of these compounds were established via spectroscopic analysis and comparison of their NMR data with the literature [17].

Kaempferol 3-*O*-α-L-rhamnopyranosyl-(1→2)-β-D-glucopyranoside (**4**): Yellow powder, HRESIMS *m*/*z* 617.1607 [M + Na]^+^ (calcd. for C_27_H_30_O_15_); ^1^H NMR (500 MHz, Acetone-*d*_6_) *δ*_H_ 8.19 (2H, d, *J* = 8.9 Hz, H-2″, 6″), 7.01 (2H, d, *J* = 8.9 Hz, H-3’, 5’), 6.56 (1H, d, *J* = 2.1 Hz, H-8), 6.30 (1H, d, *J* = 2.0 Hz, H-6), 5.17 (1H, d, *J* = 7.4 Hz, Glc H-1), 4.59 (1H, s, Rha H-1‴), 3.87–3.29 (10H, m), 1.11 (1H, d, *J* = 6.2 Hz, Rha H-6‴). ^13^C NMR (126 MHz, Acetone-*d*_6_) *δ*_C_ 178.2 (C-4), 164.5 (C-7), 162.0 (C-5), 160.3 (C-4’), 157.9 (C-9), 157.2 (C-2), 134.7 (C-3), 131.4 (C-2’, 6’), 121.4 (C-1’), 115.2 (C-3’, 5’), 104.6 (C-10), 104.5 (Glc C-1″), 101.1 (Rha C-1‴), 98.9 (C-6), 93.9 (C-8), 77.4 (Glc C-3″), 75.9 (Glc C-5″), 74.7 (Rha C-4‴), 73.1 (Rha C-2‴), 72.5 (Rha C-3‴), 71.4 (Glc C-2″), 69.8 (Rha C-5‴), 67.0 (Glc C-6″), 17.2 (Rha C-6‴). The structures of these compounds were established via spectroscopic analysis and comparison of their NMR data with the literature [18].

Rutin (**5**): Yellow powder, HRESIMS *m*/*z* 611.1770 [M + H]^+^ (calcd. for C_27_H_30_O_16_); ^1^H NMR (500 MHz, Methanol-*d*_4_) *δ*_H_ 7.65 (1H, d, *J* = 2.2 Hz, H-2’), 7.60 (1H, dd, *J* = 8.4, 2.2 Hz, H-6’), 6.85 (1H, d, *J* = 8.4 Hz, H-5’), 6.35 (1H, d, *J* = 2.1 Hz, H-6), 6.17 (1H, d, *J* = 2.1 Hz, H-8), 5.08 (1H, d, *J* = 7.5 Hz, Glc, H-1’), 4.50 (1H, d, *J* = 1.7 Hz, Rha, H-1’), 3.79 (1H, dd, *J* = 10.9, 1.5 Hz, Glc, H-6’), 3.63 (1H, dd, *J* = 3.4, 1.7 Hz), 3.56–3.21 (10H, m), 1.10 (3H, d, *J* = 6.2 Hz, Rha, H-6’). ^13^C NMR (126 MHz, Methanol-*d*_4_) *δ*_C_ 179.7 (C-4), 166.3 (C-7), 163.2 (C-5), 159.6 (C-9), 158.8 (C-2), 150.1 (C-4’), 146.1 (C-3’), 135.9 (C-3), 123.9 (C-1’), 123.4 (C-6’), 118.0 (C-5’), 116.4 (C-2’), 105.9 (C-10), 105.1 (Glc, C-1’), 102.7 (Rha, C-1’), 100.3 (C-6), 95.2 (C-8), 78.5 (Glc, C-3’), 77.5 (Glc, C-5’), 76.0 (Glc, C-2’), 74.3 (Glc, C-4’), 72.5 (Rha, C-3’), 72.4 (Rha, C-2’), 71.7 (Rha, C-4’), 70.0 (Rha, C-5’), 68.9 (Glc, C-6’), 18.2 (Rha, C-6’). The structures of these compounds were established via spectroscopic analysis and comparison of their NMR data with the literature [19].

Gingerglycolipid A (**6**): White powder, HRESIMS *m*/*z* 675.3619 [M–H]^–^ (calcd. for C_33_H_56_O_14_); ^1^H NMR (500 MHz, Methanol-*d*_4_) *δ*_H_ 5.42–5.27 (6H, m, H-9, 10, 12, 13, 15, 16), 4.60 (1H, s, H-1″), 4.29–3.45 (14H, m, H-2’, 2″, 3″, 4″, 5″, 6″, 1‴, 2‴, 3‴, 4‴, 5‴, 6‴), 2.82 (4H, t, *J* = 6.0 Hz, H-11, 14), 2.36 (2H, t, *J* = 7.5 Hz, H-2), 2.15–2.02 (2H, m, H-17), 1.62 (2H, t, *J* = 7.3 Hz, H-8), 1.36–1.26 (10H, m, H-3, 4, 5, 6, 7), 0.98 (3H, t, *J* = 7.5 Hz, H-18). ^13^C NMR (126 MHz, Methanol-*d*_4_) *δ*_C_ *175*.8 (C-1), 133.1 (C-16), 131.4 (C-15), 129.6 (C-13), 129.5 (C-12), 129.2 (C-10), 128.6 (C-9), 105.7 (C-1″), 100.9 (C-1‴), 75.0 (C-3″), 74.9 (C-5″), 72.9 (C-2″), 72.9 (C-5‴), 72.4 (C-1’), 71.8 (C-3‴), 71.4 (C-4‴), 70.6 (C-2‴), 70.5 (C-4″), 70.0 (C-2’), 68.1 (C-1″), 66.9 (C-3’), 63.1 (C-6‴), 35.3 (C-2), 31.0 (C-17), 30.7 (C-14), 30.6 (C-11), 30.5 (C-8), 28.5 (C-7), 26.9 (C-6), 26.7 (C-5), 26.3 (C-4), 21.8 (C-3), 15.0 (C-18). The structures of these compounds were established via spectroscopic analysis and comparison of their NMR data with the literature [20].

Solalyratin B (**7**): White powder, HRESIMS *m*/*z* 418.8899 [M + H]^+^ (calcd. for C_24_H_34_O_6_); ^1^H NMR (500 MHz, Methanol-*d*_4_) *δ*_H_ 7.00 (1H, d, *J* = 15.8 Hz, H-7′), 6.44 (1H, d, *J* = 15.8 Hz, H-8′), 5.94 (1H, s, C-2′), 5.75 (1H, s, C-2), 4.26–4.15 (1H, m, C-6), 2.61 (1H, d, *J* = 17.0 Hz, C-6′), 2.42 (1H, dd, *J* = 13.5, 2.7 Hz, C-5), 2.31 (3H, s, C-10′), 2.02–1.97 (1H, m, C-7), 1.90 (3H, d, *J* = 1.30 Hz, C-11′), 1.76 (3H, s, C-11), 1.53 (1H, dd, *J* = 14.4, 3.7 Hz, C-7), 1.47 (2H, s, C-9), 1.29 (3H, s, C-10), 1.07 (3H, s,C-12′), 1.02 (3H, s, C-13′). ^13^C NMR (126 MHz, Methanol-*d*_4_) *δ*_C_ 200.7 (C-9′), 200.4 (C-1′), 185.7 (C-1), 174.5 (C-3), 148.4 (C-7′), 131.7 (C-8′), 128.0 (C-2′), 113.3 (C-2), 89.0 (C-4), 80.0 (C-4′), 67.3 (C-6), 50.5 (C-6′), 48.0 (C-7), 46.5 (C-5), 42.7 (C-5′), 37.2 (C-8), 31.0 (C-10), 27.6 (C-10′), 27.4 (C-11), 27.0 (C-9), 24.7 (C-13′), 23.5 (C-1 2′), 19.2 (C-11′). The structures of these compounds were established via spectroscopic analysis and comparison of their NMR data with the literature [21].

(+)-Syringaresinol (**8**): White columnar crystals, HRESIMS *m*/*z* 419.1768 [M + H]^+^ (calcd. for C_22_H_26_O_8_); ^1^H NMR (500 MHz, Methanol-*d*_4_) *δ*_H_ 6.66 (4H, s, H-2’, H-6,’ H-2″, H-6″), 4.71 (2H, d, *J* = 4.5 Hz, H-2, H-6), 4.26 (2H, dd, *J* = 9.0, 7.0 Hz, H-4a, H-8a), 3.88 (2H, dd, *J* = 9.0, 4.0 Hz, H-4b, H-8b), 3.84 (12H, s, 3’, 5’, 3″, 5″-OCH_3_), 3.16–3.11 (2H, m, H-1, H-5). ^13^C NMR (126 MHz, Methanol-*d*_4_) *δ*_C_ 149.5 (C-3’, 5’, 3″, 5″), 136.3 (C-4’, 4″), 133.3 (C-1’, 1″), 104.6 (C-2’, 6’, 2″, 6″), 87.8 (C-2, 6), 72.9 (C-4, 8), 56.9 (C-3’, 5’, 3″, 5″-OMe), 55.7 (C-1, 5). The structures of these compounds were established via spectroscopic analysis and comparison of their NMR data with the literature [22].

(+)-Mediastinal (**9**): White amorphous powder, HRESIMS *m*/*z* 389.1628 [M + H]^+^ (calcd. for C_21_H_24_O_7_); ^1^H NMR (500 MHz, Methanol-*d*_4_) *δ*_H_ 6.95 (1H, d, *J* = 1.7 Hz, H-2), 6.82 (1H, dd, *J* = 1.7, 8.1 Hz, H-6), 6.77 (1H, d, *J* = 8.1 Hz, H-5), 6.66 (2H, s, H-2′, 6′), 4.72 (2H, m, H-7, 7′), 4.30–4.20 (2H, m, H-9, 9′), 3.86 (3H, s, 3-OMe), 3.85 (6H, s, 3′, 5′-OMe), 3.16–3.13 (2H, m, H-8, 8′). ^13^C NMR (126 MHz, Methanol-*d*_4_) *δ*_C_ 149.5 (C-3′, 5′), 149.3 (C-3), 147.5 (C-4), 136.4 (C-4′), 134.0 (C-1), 133.3 (C-1′), 120.3 (C-6), 116.3 (C-5), 111.2 (C-2), 104.7 (C-2′, 6′), 87.9 (C-7′), 87.7 (C-7), 72.9 (C-9′), 72.8 (C-9), 57.0 (3′, 5′-OMe), 56.6 (3-OMe), 55.7 (C-8), 55.5 (C-8′). The structures of these compounds were established via spectroscopic analysis and comparison of their NMR data with the literature [23].

(−)-Pinoresinol (**10**): Colorless crystals, HRESIMS *m*/*z* 341.1444 [M–H_2_O + H]^+^ (calcd. for C_20_H_22_O_6_); ^1^H NMR (500 MHz, Methanol-*d*_4_) *δ*_H_ 6.95 (2H, d, *J* = 1.9 Hz, H-2, 2′), 6.82 (2H, dd, *J* = 8.2, 1.9 Hz, H-6, 6′), 6.77 (2H, d, *J* = 8.1 Hz, H-5, 5′), 4.71 (1H, d, *J* = 4.7 Hz, H-7, 7′), 4.24 (1H, dd, *J* = 9.1, 6.9 Hz, H-9a, 9a′), 3.86 (6H, s, 3, 3′-OMe), 3.85 (2H, s, H-9b, 9b′), 3.17–3.12 (2H, m, H-8, 8′). ^13^C NMR (126 MHz, Methanol-*d*_4_) *δ*_C_ 149.3 (C-3, 3′), 147.5 (C-4, 4′), 133.9 (C-1, 1′), 120.2 (C-6, 6′), 116.3 (C-5, 5′), 111.1 (C-2, 2′), 87.7 (C-7, 7′), 72.8 (C-9, 9′), 56.6 (2×OMe), 55.5 (C-8, 8′). The structures of these compounds were established via spectroscopic analysis and comparison of their NMR data with the literature [24].

(+)-*epi*-Syringaldehyde (**11**): Colorless solid, HRESIMS *m*/*z* 441.1581 [M + Na]^+^ (calcd. for C_22_H_26_O_8_); ^1^H NMR (500 MHz, Methanol-*d*_4_) *δ*_H_ 6.95 (2H, d, *J* = 1.9 Hz, H-2, 2′), 6.82 (2H, dd, *J* = 8.2, 1.9 Hz, H-6, 6′), 6.77 (2H, d, *J* = 8.1 Hz, H-5, 5′), 4.71 (1H, d, *J* = 4.7 Hz, H-7, 7′), 4.24 (1H, dd, *J* = 9.1, 6.9 Hz, H-9a, 9a′), 3.86 (6H, s, 3, 3′-OMe), 3.85 (2H, s, H-9b, 9b′), 3.17–3.12 (2H, m, H-8, 8′). ^13^C NMR (126 MHz, Methanol-*d*_4_) *δ*_C_ 149.3 (C-3, 3′), 147.5 (C-4, 4′), 133.9 (C-1, 1′), 120.2 (C-6, 6′), 116.3 (C-5, 5′), 111.1 (C-2, 2′), 87.7 (C-7, 7′), 72.8 (C-9, 9′), 56.6 (2×OMe), 55.5 (C-8, 8′). The structures of these compounds were established via spectroscopic analysis and comparison of their NMR data with the literature [25].

ω-Hydro-xypropioguaiacone (**12**): Colorless oily substance, HRESIMS *m*/*z* 195.0620 [M–H]^−^ (calcd. for C_10_H_12_O_4_); ^1^H NMR (500 MHz, Methanol-*d*_4_) *δ*_H_ 7.58 (1H, dd, *J* = 8.3, 2.0 Hz, H-6′), 7.43 (1H, d, *J* = 2.0 Hz, H-2′), 6.92 (1H, d, *J* = 8.3 Hz, H-5′), 4.32 (3H, s, OMe) 3.93 (2H, t, *J* = 6.1 Hz, H-3), 3.16 (2H, t, *J* = 6.2 Hz, H-2). ^13^C NMR (126 MHz, Methanol-*d*_4_) *δ*_C_ 200.2 (C-1), 154.2 (C-3′),149.7 (C-4′), 131.0 (C-1′),125.3 (C-6′), 116.4 (C-5′), 112.4 (C-2′), 59.5 (C-3), 56.9 (OMe), 42.2 (C-2). The structures of these compounds were established via spectroscopic analysis and comparison of their NMR data with the literature [26].

Vanillic acid (**13**): Colorless needle crystals, HRESIMS *m*/*z* 169.0491 [M+H]^+^ (calcd. for C_8_H_8_O_4_); ^1^H NMR (500 MHz, Methanol-*d*_4_) *δ*_H_ 7.58 (1H s Ar-H), 7.53 (1H, d, *J* = 8.2 Hz, Ar-H), 6.80 (1H, d, *J* = 8.2 Hz, Ar-H), 3.89 (3H, s, -OMe). ^13^C NMR (126 MHz, Methanol-*d*_4_) *δ*_C_ 172.2 (C-7), 151.5 (C-3), 148.4 (C-2), 124.8 (C-5), 120.4 (C-6) 115.5 (C-1), 113.9 (C-4), 56.3 (OMe). The structures of these compounds were established via spectroscopic analysis and comparison of their NMR data with the literature [27].

4-Hydroxybenzaldehyde (**14**): Colorless oily substance, HRESIMS *m*/*z* 121.0345 [M+H]^+^ (calcd. for C_7_H_6_O_2_); ^1^H NMR (500 MHz, Methanol-*d*_4_) *δ*_H_ 9.76 (1H, s, COH), 7.77 (2H, d, *J* = 8.6 Hz, H-2, 6), 6.91 (2H, d, *J* = 8.6 Hz, H-3, 5). ^13^C NMR (126 MHz, Methanol-*d*_4_) *δ*_C_ 192.3 (-COH), 165.2 (C-4), 133.0 (C-2, 6), 129.6 (C-1), 116.5 (C-3, 5). The structures of these compounds were established via spectroscopic analysis and comparison of their NMR data with the literature [28].

2-Methoxyhydroquinone (**15**): White powder, HRESIMS *m*/*z* 141.3576 [M + H]^+^ (calcd. for C_7_H_8_O_3_); ^1^H NMR (500 MHz, Methanol-*d*_4_) *δ*_H_ 7.59 (1H, d, *J* = 1.9 Hz, H-3), 7.53 (1H, dd, *J* = 8.2, 1.9 Hz, H-6), 6.79 (1H, d, *J* = 8.2 Hz, H-5), 3.89 (3H, s, 2-OMe). ^13^C NMR (126 MHz, Methanol-*d*_4_) *δ*_C_ 150.5 (C-4), 147.8 (C-2), 128.3 (C-1), 124.1 (C-6), 114.9 (C-5), 113.47 (C-3), 55.8 (2-OMe). The structures of these compounds were established via spectroscopic analysis and comparison of their NMR data with the literature [29].

### 2.6. Antioxidant Activity

The ABTS free radical scavenging capacity of all isolated compounds was measured, and the procedure followed a method with minor modifications [30]. The experiment was conducted using a 96-well plate, with each well containing a total volume of 210 µL. Equal volumes of ABTS solution (7 mM) and potassium persulfate solution (5 mM) were thoroughly mixed and allowed to react at room temperature for 12 h in the absence of light to generate the ABTS radical cation. The mixture was then diluted with anhydrous methanol to achieve an absorbance value of about 0.7 ± 0.02 units at 734 nm. Then, 180 µL of ABTS working solution was added to each well, followed by 30 µL samples of varying concentrations (0.5, 0.1, 0.05, 0.01 µg/mL) were dissolved and diluted with DMSO. After thorough mixing, the samples were incubated at room temperature for 6 min in a light-free environment. Subsequently, the absorbance values of each well were measured at 734 nm using a microplate reader, and the results were obtained from a minimum of three independent experiments. Ascorbic acid was employed as the positive control. DMSO was used to replace the sample solution as a blank and absolute methanol was used to replace the ABTS solution as a control. All tests were performed in triplicate, and the obtained results were processed by analysis of variance (ANOVA) with 95% confidence (*p* ≤ 0.05).

The percentage radical cation scavenging rate (%) of each test sample for ABTS was calculated as follows (1):

(1) Radical cation scavenging rate (%) = [A _blank_ − (A _sample_ − A _control_)] / A _blank_ × 100%

### 2.7. Antimicrobial Activity

The antibacterial activity capacity of all isolated compounds was measured, and the procedure followed a method with minor modifications [31,32]. Antimicrobial drug mother liquor preparation: dissolve the drug with DMSO, and its mass concentration was 0.5 mg/mL. Preparation of the bacterial solution to be tested: Thaw the frozen bacteria from a −80 °C low-temperature storage box at room temperature, and sterilize the Nutrient Broth (NB) medium in a 37 °C shaking bed for overnight culture. Then take 2 mL of the overnight culture of the bacterial solution and inoculate it into the NB medium. Incubate it at 37 °C until A_600_ = 0.5, and then dilute it 100 times with the NB medium. Micro broth dilution method: Take a sterile 96-well plate, add 75 μL of the NB medium dilution solution to the A2–A11 wells, and add 75 μL of the drug solution to the A1–A2 wells. Take 75 μL of the mixture from the A2 wells to the A3 wells, 75 μL of the mixture from the A3 wells to the A4 wells, and so on up to the A10 wells. Take up 75 μL of the mixture to the A10 wells, discard it, and add 75 μL of 5% DMSO to the 12 wells. Add 75 μL of the bacterial solution to well A12 and mix well. The inoculated 96-well plates were incubated at 37 °C for 12 h. After inoculation, the 96-well plates were incubated at 37 °C for 14 h to observe the growth. The minimum inhibitory concentration (MIC) was calculated by measuring A_600_ with an enzyme marker. All tests were performed in triplicate, and the obtained results were processed by (ANOVA) with 95% confidence (*p* ≤ 0.05).

## 3. Results and Discussion

### 3.1. Antioxidant Activity

In the ABTS assay, the antioxidant activity is measured as the ability of test compounds to decrease the color by reacting directly with the radical ABTS [33]. The antioxidant activities of all the isolates were assessed in vitro using the ABTS assay method, and the corresponding results are presented in Table 2. The results of these experiments demonstrated that compounds **5**, and **8**–**11** exhibited superior antioxidant activity compared to the positive drug ascorbic acid, while the activities of compounds **7**, **13,** and **15** were comparable to that of ascorbic acid. The other compounds had weaker or no significant antioxidant activities. This finding once again confirms that flavonoids [34,35,36], terpenoids [37,38], lignans [39,40,41], and phenolic acid [42] compounds have good antioxidant activities. The compounds **1**–**5** are all flavonoid glycosides; however, compound **5** demonstrates significantly superior antioxidant activity in comparison to compounds **1**–**4**. An analysis of the structure–activity relationship for these five flavonoid glycosides reveals a direct correlation between the free radical scavenging activity and the number of hydroxyl groups on the B ring. Increasing the number of hydroxyl groups on the B ring enhances the free radical scavenging activity. Moreover, it is noteworthy that C–3 possesses an alcohol hydroxyl group, which exhibits greater stability and reduced susceptibility to electron loss compared to a phenol hydroxyl group. This characteristic contributes to increased water solubility of compounds without significantly affecting their antioxidant activity. These findings are consistent with the previous literature analyses regarding the conformational relationship between flavonoid antioxidant activities [43,44,45]. Meanwhile, the antioxidant activities of compounds **1**–**4** also differed, which was mainly related to the number and position of hydroxyl groups and the spatial site resistance of glycosides [46]. The lignan compounds **8**–**11** possess two phenolic hydroxyl groups, and their antioxidant activities exhibit minimal variation among each other. This similarity may be attributed to the positive impact of the methoxy group, located adjacent to the phenolic hydroxyl group, on the antioxidant activity of lignans in the power supply [47]. Compounds **12**–**15** are phenolic acids derived from hydroxybenzoic acid, with compound **14** exhibiting significantly lower antioxidant activity compared to compounds **12**–**13** and **15**. Furthermore, the introduction of o-hydroxy and o-methoxy groups enhances the antioxidant activity of phenolic acids [48]. The findings suggest that flavonoids, phenols, lignans, and terpenoids are the primary antioxidant secondary metabolites in *C. fascicularis*. Due to the limited quantity of certain compounds and their inadequate initial concentration of 500 µg/mL for effective antioxidant activity, experiments were not conducted on these compounds considering the requirement for subsequent sequential activity determination. The utilization of only ABTS as an indicator for assessing antioxidants may not comprehensively reflect the complete antioxidant activity of compounds.

### 3.2. Antibacterial Activity

The utilization of botanical extracts as natural antimicrobial agents in the food industry is an emerging trend [49]. The antimicrobial activity testing of the samples was performed against microorganism strains from the laboratory collection. The Gram-positive bacteria used for the tests were *Staphylococcus aureus* (ATCC 6538). The Gram-negative bacteria were *Escherichia coli* (ATCC 6538) and *Pseudomonas aeruginosa* (CGMCC 1.10712). The antimicrobial activity of *C. fascicularis* has not been reported, but the antimicrobial activity of researchers in *Camellia* proved that *Camellia* has good antimicrobial activity [50,51,52]. The findings provide additional evidence to substantiate the potent antibacterial activity of secondary metabolites derived from *C. fascicularis*.

As shown in Table 3, the results of the antimicrobial activity test showed that compounds **1**–**15** all showed some degree of inhibition against *E. coli* at 125–250 µg/mL, with compound **7** showing better antimicrobial activity than the other compounds, but still lower than the positive control drugs penicillin (MIC 31.25 µg/mL) and tetracycline (MIC 7.81 µg/mL). Compounds **3**, **5**–**9**, **11**, and **14**–**15** showed a certain degree of inhibition of *S. aureus* at 125–250 µg/mL, which was still weaker compared with the positive control drug. The antibacterial activity of compounds **5**, **7**, **9**, **11**, and **13** against *P. aeruginosa* was comparable to that of the positive control drug tetracycline (MIC 62.50 µg/mL) and superior to that of penicillin (MIC 125.00 ug/mL), whereas the antibacterial activity of compound **15** against *P. aeruginosa* showed antimicrobial activity comparable to the positive control drug penicillin (MIC 125.00 µg/mL) and weaker than tetracycline (MIC 62.50 µg/mL). The findings suggest that flavonoids, phenolics, and terpenoids serve as the primary secondary metabolites responsible for the antimicrobial activity in *C. fascicularis*. The MIC data of 250.00 μg/mL in antibacterial activity indicates a range of true valuest from 125.00 to 500.00 μg/mL. It is important to note that this method provides an approximation rather than an absolute accurate value for the MIC. If it were possible to provide microstructures of different bacteria at optimal inhibitory concentrations and scanning electron microscopy images, it would further support our study; unfortunately, all compounds were completely consumed during the series of bioactivity assays.

The structures of the known compounds were defined as tsubakioside A (**1**), kaempferol 3-*O*-rutinoside (**2**), camelliaside B (**3**), kaempferol 3-*O*-α-L-rhamnopyranosyl-(1→2)-β-D-glucopyranoside (**4**), rutin (**5**), gingerglycolipid A (**6**), solalyratin B (**7**), (+)-syringaresinol (**8**), (+)-mediastinal (**9**), (–)-pinoresinol (**10**), (–)-*epi*-syringaldehyde (**11**), ω-hydro-xypropioguaiacone (**12**), vanillic acid (**13**), 4-hydroxybenzaldehyde (**14**), and 2-methoxyhydroquinone (**15**), respectively.

The presence of compounds (**1**–**5**) in the Theaceae family has been previously demonstrated through chemical studies [17,53,54], highlighting the abundant and diverse flavonoid content in *Camellia.* Compound **6** was initially derived from plants belonging to the genus *Ginger* in the Zingiberaceae family. It serves as a crucial metabolite involved in various physiological processes, including germination, growth, flowering, senescence, and fruit ripening in higher plants. Additionally, it plays a significant role in evaluating the effects of glucose-lowering [55,56,57,58]. Compound **7** is a tetraterpene with notable anti-inflammatory and anti-complementary activities. Moreover, this study demonstrates its remarkable antioxidant and antibacterial properties [21,59]. In addition, lignans (**8**–**11**) have been recognized as bioactive components with applications in the pharmaceutical and nutritional industries [60,61]. Compounds (**12**–**15**), as phenolic acids, are widely found in Theaceae plants [62,63].

## 4. Conclusions

Secondary metabolites investigation on the leaves of *C. fascicularis* afforded 15 compounds, including 5 flavonoids (**1**–**5**), a galactosylglycerol derivative (**6**), a terpenoid (**7**), 4 lignans (**8**–**11**), and 4 phenolic acids (**12**–**15**). Compounds **6**–**7** and **9**–**12** were isolated from the genus *Camellia* for the first time. The remaining compounds were also isolated from this plant for the first time. The antioxidant and antimicrobial activities of the isolated and obtained compounds were determined. The evaluation of antioxidant and antimicrobial activities demonstrated that compounds **5** and **8**–**11** exhibited superior antioxidant activity compared to the positive control drug ascorbic acid. Compounds **7**, **13**, and **15** displayed similar activity to ascorbic acid. In terms of antibacterial activity against *P. aeruginosa*, compounds **5**, **7**, **9**, **11**, and **13** showed comparable MIC values to the positive control drug tetracycline at a concentration of 62.50 µg/mL. Additionally, other secondary metabolites inhibited *E. coli* and *S. aureus* at concentrations ranging from 125–250 µg/mL. The experimental results suggested that flavonoids, phenols, lignans, and terpenoids are the main secondary metabolites of antioxidant and antibacterial activities in *C. fascicularis*. The results of the study enriched the variety of secondary metabolites of *C. fascicularis*, laid the foundation for further research on the pharmacological efficacy and biological activity of this plant, and also provided a reference and theoretical basis for the development and utilization of this plant resource.

## Figures and Tables

**Table 1:** Weight in (Fr. A–I) of *C. fasciculata*.

Fractions (g)								
A	^1^ B*	D	E	F	G	H	I	Fr. II–III
6.16	2.61	4.52	7.16	5.63	9.53	2.81	5.73	44.15/45.00

“^1^ B*” was obtained by combining the B and C fractions.

**Table 2:** Antioxidant activities of chemical constituents **1**–**15** in *C. fascicularis*.

Compound	ABTS^+ B^ Assay (%)			
	500 µg/mL	100 µg/mL	50 µg/mL	10 µg/mL
1	49.84 ± 1.88 ^e^	-	-	-
2	43.23 ± 2.54 ^f^	-	-	-
3	42.73 ± 1.81 ^f^	-	-	-
4	66.40 ± 1.17 ^d^	-	-	-
5	99.07 ± 0.49 ^a^	98.75 ± 0.22 ^a^	85.90 ± 1.0 ^b^	15.59 ± 3.21 ^f^
6	20.12 ± 1.09 ^i^	-	-	-
7	90.81 ± 0.90 ^b^	70.08 ± 3.86 ^b^	39.61 ± 2.73 ^e^	-
8	99.85 ± 0.65 ^a^	99.89 ± 0.14 ^a^	98.31 ± 1.22 ^a^	21.59 ± 0.14 ^cde^
9	98.66 ± 0.84 ^a^	99.20 ± 0.74 ^a^	97.88 ± 1.11 ^a^	36.61 ± 0.42 ^a^
10	99.48 ± 0.46 ^a^	99.82 ± 0.24 ^a^	86.74 ± 1.54 ^b^	26.25 ± 0.53 ^b^
11	99.40 ± 0.61 ^a^	99.32 ± 0.27 ^a^	98.36 ± 0.34 ^a^	22.28 ± 2.72 ^cd^
12	100.16 ± 0.90 ^a^	99.47 ± 0.76 ^a^	87.82 ± 1.38 ^b^	19.91 ± 1.54 ^de^
13	87.22 ± 1.23 ^c^	63.87 ± 2.13 ^c^	54.63 ± 2.14 ^d^	-
14	40.18 ± 0.69 ^g^	-	-	-
15	97.69 ± 3.54 ^a^	58.11 ± 2.46 ^d^	44.02 ± 4.27 ^e^	18.54 ± 1.44 ^ef^
^A^ ascorbic acid	-	99.85 ± 0.03 ^a^	61.78 ± 0.69 ^c^	25.00 ± 2.06 ^bc^

“^A^” positive control; “^B^” inhibition ratio, “-” indicates that the experiment was not performed. Values accompanied by different letters are significantly different (*p* ≤ 0.05).

**Table 3:** Antibacterial activity of chemical components **1**–**15** in *C. fascicularis*.

Compound	MIC ^b^ µg/mL		
	*E. coli*	*S*. *aureus*	*P*. *aeruginosa*
1	250.00	-	-
2	250.00	-	-
3	250.00	250.00	-
4	250.00	-	-
5	250.00	250.00	62.50
6	250.00	250.00	-
7	125.00	250.00	62.50
8	250.00	250.00	-
9	250.00	250.00	62.50
10	250.00	-	-
11	250.00	125.00	62.50
12	250.00	-	-
13	250.00	-	62.50
14	250.00	250.00	-
15	250.00	250.00	125.00
^a^ Penicillin	31.25	31.25	125.00
^a^ Tetracycline	7.81	15.63	62.50

“^a^” positive control; “^b^” minimum inhibitory concentration. “-” indicates that the experiment was not performed.

## Data Availability

The data presented in this study are available upon request from the corresponding author.
